# Study on the Effects of Individualized Nutritional Intervention on Pregnancy Outcome and Neonatal Immune Function in Patients with Gestational Diabetes Mellitus

**DOI:** 10.1155/2022/3246784

**Published:** 2022-01-05

**Authors:** Xiaofeng Zhang, Yudan Wu, Liye Miao

**Affiliations:** Department of Obstetrics, High-Tech District, Fourth Hospital of Shijiazhuang City, Hebei, China

## Abstract

**Objective:**

To study the effects of individualized nutritional intervention on pregnancy outcome and neonatal immune function in patients with gestational diabetes mellitus (GDM).

**Methods:**

A retrospective analysis was conducted on 100 GDM patients from the obstetrics and gynecology department of our institute between February 2019 and February 2020. The patients were allocated into the control group given regular intervention and the experimental group given individualized nutritional intervention according to different intervention measures, with 50 cases in each group. The comparison was carried out for patients in the two groups with regard to their modality of delivery, neonatal health, their plasma glucose in fasting state, 2 h after eating, and before bedtime; glycohemoglobin at 8 months of pregnancy, at 9 months of pregnancy, during labor, and 1 month after delivery; their complications; and neonatal CD3^+^, CD4^+^, and CD8^+^ levels.

**Results:**

The experimental group outperformed the control group in terms of the spontaneous delivery rate, the number of healthy neonates, and neonatal CD3^+^, CD4^+^, and CD8^+^ levels (*P* < 0.05). The plasma glucose in fasting state, 2 h after eating, and before bedtime; the glycohemoglobin at 8 months of pregnancy, at 9 months of pregnancy, during labor, and 1 month after delivery; and the incidence of complications of the experimental group were significantly lower than those of the control group (*P* < 0.05).

**Conclusion:**

Individualized nutritional intervention increases the rate of spontaneous delivery in GDM patients, enhances neonatal immune function, stabilizes plasma glucose, and reduces complications.

## 1. Introduction

Gestational diabetes mellitus (GDM) is a disease specific to gravidas during pregnancy. It is defined as diabetes diagnosed when blood sugar levels remain normal in the progestational stage and thereafter rise during gestation in the affected population. During pregnancy, pregnant women will have abnormal blood glucose metabolism due to the placenta secreting hormones that antagonize insulin; additionally, the impaired nutritional metabolism can also easily lead to energy metabolism and increased blood sugar [1–3]. Patients with gestational diabetes run a much greater risk of intrapartum hemorrhage and premature rupture of membranes than healthy gravidas, which severely threatens the health of the mother and fetus. Patients with gestational diabetes have reported a low rate of spontaneous delivery, which may predispose them to neonatal asphyxia and result in poor delivery outcomes [4–6]. In addition, oral medication for blood sugar control in diabetics increases the risk of fetal malformations. Therefore, the control and stabilization of blood glucose levels without medication during pregnancy are a major concern for gestational diabetic patients [2, 7, 8]. Adequate nutritional status is essential to health, and pregnant women are susceptible to impaired access to nutritious diets, and increased medication use increases the risk of nutritional disorders. According to epidemiological statistics, the prevalence of malnutrition in pregnant women with gestational diabetes ranges from 15% to 60% [1, 2]; consequently, early detection of malnutrition and timely interventions serve to improve the health status of pregnant women with gestational diabetes and reduce the occurrence of adverse events. Individualized nutritional interventions are used to help patients achieve nutritional homeostasis and reduce the incidence of overnutrition or malnutrition. At present, clinical interventions for GDM are mainly based on scientific diet. The individualized diet plans are formulated based on personal condition, body mass, exercise habits, and other basic information in terms of eating time and amount of eating. It was found that despite the scientific diet plan, it still lacks management structure, thereby leading to incomplete implementation and unsatisfactory clinical effect; moreover, despite a certain amount of daily activities, its arbitrariness also results in little effect on the disease [2]. In response to the above-mentioned problems, this study reviewed the literature; summarized clinical experience; formulated a scientific diet as the mainstay, with exercise training as an adjuvant method; and established a professional team to conduct in-depth training and real-time supervision to maximize the intervention plan. In this study, individualized nutritional intervention and regular intervention were conducted in pregnant patients with diabetes. The comparison was carried out for patients in the two groups with regard to their modality of delivery; neonatal health; their plasma glucose in fasting state, 2 h after eating, and before bedtime; glycohemoglobin at 8 months of pregnancy, at 9 months of pregnancy, during labor, and 1 month after delivery; their complications; and neonatal CD3^+^, CD4^+^, and CD8^+^ levels, with an aim to analyze the effects of individualized nutritional intervention for gravidas and newborns. Specific studies are described below.

## 2. Materials and Methods

### 2.1. General Data

A retrospective analysis was conducted on 100 GDM patients from the obstetrics and gynecology department of our institute between February 2019 and February 2020. They were allocated into two groups (a control group and an experimental group) according to different intervention measures, with 50 cases in each group. Patients in the experimental group (given individualized nutritional intervention) and the control group (given routine nutritional intervention) are aged 22-31 years and 21-35 years, respectively. The two groups showed no significant difference in baseline data (*P* > 0.05), as seen in Table 1. This study was approved by the ethics committee of our hospital; the ethics approval number is 2018-12-22.

### 2.2. Inclusion/Exclusion Criteria

#### 2.2.1. Inclusion Criteria

1. Those who conformed to clinical manifestations of diabetes in pregnancy, diabetes or reduced glucose tolerance for the first time during pregnancy (excluding patients diagnosed with diabetes before pregnancy) with fasting glucose greater than or equal to 5.1 mmol/L, polydrinking, polyphagia, polyuria, and malaise
2. Age of gravidas > 18 years old
3. Those without other organic diseases
4. Those with no prior history of drug allergy and drug abuse and bad addiction
5. Our institute Ethics Committee approved the study, and patients voluntarily participated in the study and provided informed consent

#### 2.2.2. Exclusion Criteria

1. Pregnant women with twin pregnancies
2. Age of gravida ≥ 40 years
3. Patients who were diagnosed with diabetes mellitus before gestation
4. Pregnant women with premature birth or miscarriage, as well as patients with severe cardiovascular diseases and those in need of strict bed rest

### 2.3. Methods

The treatment was given to patients in both groups within 28 gestational weeks after diagnosis and continued until 36 weeks. Health education was provided to the control group, including the characteristics and hazards of gestational diabetes, the significance and practice of diet control and exercise therapy, the importance of blood glucose testing and daily targets for blood glucose control, knowledge of insulin pharmacology, and skin care, and the psychiatrist relieves the mother's mood. In addition, nutrition consultation by a full-time nutrition nurse was set up in the hospital to answer questions from the patients and their families in a timely manner. Patients were informed of the nutritional standards for gestational diabetes and were instructed on appropriate exercise and dietary control to stabilize their blood glucose. Moreover, caregivers helped patients develop appropriate diet regimes, exercise plans, and daily schedules and conducted regular follow-up visits to inspect their blood glucose and glycated hemoglobin levels.

Individualized nutritional intervention was carried out for patients in the experimental group. The dietitian calculated the daily calorie requirement based on the standard weight of pregnant women and formulated a personalized dietary scheme according to their weight, blood sugar, and gestational weeks. The nurse in charge assisted the dietitian in diet education and guidance. For patients with poor self-control, nurses supervised their diet and strengthened health education to raise awareness among the patients and their families about gestational diabetes and the importance of strict implementation of the dietary programs. Pregnant women and their families were instructed to master the food exchange method to help pregnant women develop healthy eating habits and establish a reasonable diet structure to achieve blood sugar control. Their nutrition status and plasma glucose levels were tested periodically to ensure balanced nutrition. Individuals' diet schedules were developed depending on their nutrition status, focusing on the intake of coarse fiber food, protein, and vitamins and controllable ingestion of high-sugar and high-fat meals, to circumvent the accumulation of sugar and fat. For nutritional evaluation, after clarifying the patient's gestational age, family history, age, weight, exercise, diet, blood sugar level, and other basic conditions, the plan is formulated,total daily caloric intake = standard weight × energy coefficient, patients at second-trimester pregnancies +200 kcal per day. The nutrient and energy of the patients are proportioned by the food exchange method, 6 meals/d, with soybean oil as the daily oil, vegetables 500 g/d, and low-sugar fruits (<200 g) between meals [9]. In addition, patients were instructed to perform an appropriate exercise routine, prioritizing walking. Exercise plan mainly includes gravida gymnastics, walking, and aerobic exercise 30 min after meal, 30 min/d; it is appropriate for pregnant women to show slight fatigue [10]. Health education was conducted to understand gestational diabetes and to raise their health awareness. The nurse in charge formulated an appropriate exercise program with the pregnant women with gestational diabetes based on their condition and blood glucose, gestational week, height, and weight. Exercises mainly include walking, tai chi, maternity exercises, and yoga. The duration of exercise was normally limited to 20-40 minutes, preferably without causing contractions. Family members or nurses were required to accompany the patients during exercise in case of accidents.

### 2.4. Observation Indicators

The comparison was carried out for patients in the two groups with regard to their modality of delivery; neonatal health; their plasma glucose in fasting state, 2 h after eating, and before bedtime; glycohemoglobin at 8 months of pregnancy, at 9 months of pregnancy, during labor, and 1 month after delivery; their complications; and neonatal CD3^+^, CD4^+^, and CD8^+^ levels.

All GDM patients in both groups were followed up, and no one was lost.

### 2.5. Statistical Analysis

The SPSS 20.0 was used for data analysis, and GraphPad Prism7 (GraphPad Software, San Diego, USA) was utilized to visualize the data. Data included in the study consisted of counting data and measurement data. Measurement data were expressed as ($\bar{x}\pm s$) and analyzed using the *t*-test. Counting data were expressed as [*n* (%)] and analyzed using the chi-square test. *P* < 0.05 was considered statistically significant.

## 3. Results

### 3.1. Modality of Delivery

**Figure 1:**
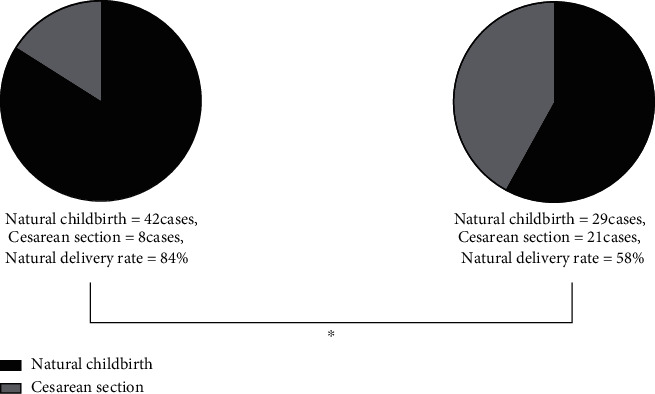
Comparison of the modality of delivery between the two groups. (a) Shows the labor outcome of the experimental group, in which 42 cases were delivered spontaneously and 8 cases were delivered by cesarean section, and the spontaneous delivery rate was up to 84%. (b) Shows the labor outcome of the control group, in which 29 cases were delivered spontaneously and 21 cases were delivered by cesarean section, and the spontaneous delivery rate was up to 58%. ∗ indicates the comparison of labor outcomes between the two groups (*X*^2^ = 8.21, *P* = 0.004), and comparison results achieved statistical significance.

It was revealed by the comparison of two modalities of delivery that a higher rate of spontaneous delivery onset was found in the experimental group versus the control group (*P* < 0.05) (Figure 1).

### 3.2. Neonatal Outcomes

It was demonstrated through comparison that there were more healthy newborns in the experimental group with fewer cases of fetal macrosomia as compared to the control group (*P* < 0.05) (Table 2).

### 3.3. Blood Glucose Levels at Different Time Points

**Figure 2:**
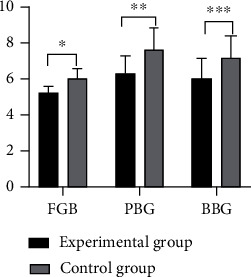
Comparison of blood glucose levels at different time points between the two groups. Notes: FBG: fasting blood glucose; PBG: postprandial blood glucose; BBG: bedtime blood glucose. The horizontal coordinate indicates blood glucose in fasting state, 2 h after eating, and before bedtime from left to right, and the vertical coordinate indicates blood glucose levels. ∗ indicates fasting blood glucose (5.21 ± 0.38 mmol/L) in the experimental group versus fasting blood glucose (6.03 ± 0.55 mmol/L) in the control group (*t* = 8.67, *p* < 0.001), and comparison results had statistical significance. ∗∗ indicates blood glucose 2 h after eating (6.28 ± 1.00 mmol/L) in the experimental group versus blood glucose 2 h after eating (7.61 ± 1.21 mmol/L) in the control group (*t* = 5.99, *P* < 0.001), and comparison results were proven to have statistical significance. ∗∗∗ indicates blood glucose before bedtime (6.08 ± 1.04 mmol/L) in the experimental group versus blood glucose before bedtime (7.16 ± 1.24 mmol/L) in the control group (*t* = 4.72, *P* < 0.001), and comparison results exhibited statistical significance.

It was displayed through comparison that patients in the experimental group possessed markedly lower levels of blood glucose in the fasting state, 2 h after eating, and before bedtime than the control group (*P* < 0.05) (Figure 2).

### 3.4. Glycohemoglobin at 8 Months of Pregnancy, at 9 Months of Pregnancy, during Labor, and 1 Month after Delivery

**Figure 3:**
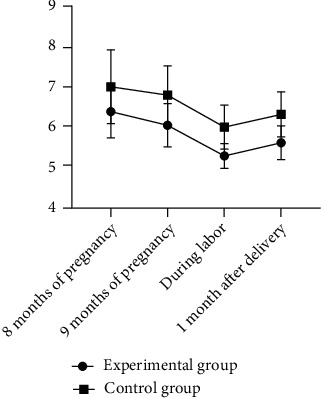
Comparison of the glycohemoglobin at 8 months of pregnancy, at 9 months of pregnancy, during labor, and 1 month after delivery between the two groups. Note: the horizontal coordinate indicates 8 months of pregnancy, 9 months of pregnancy, during labor, and 1 month after delivery from left to right. The vertical coordinate indicates the glycohemoglobin index. This figure shows glycohemoglobin at 8 months of pregnancy (6.39 ± 0.66%) in the experimental group versus glycohemoglobin at 8 months of pregnancy (7.01 ± 0.92%) in the control group (*t* = 3.87, *P* < 0.001), which exhibited statistical significance; glycohemoglobin at 9 months of pregnancy (6.05 ± 0.54%) in the experimental group versus glycohemoglobin at 9 months of pregnancy (6.80 ± 0.73%) in the control group (*t* = 5.84, *P* < 0.001), which was proven to have statistical significance; glycohemoglobin during labor (5.28 ± 0.31%) in the experimental group versus glycohemoglobin during labor (6.00 ± 0.55%) in the control group (*t* = 8.06, *P* < 0.001), which reached statistical significance; and glycohemoglobin 1 month after delivery (5.61 ± 0.42%) in the experimental group versus glycohemoglobin 1 month after delivery (6.32 ± 0.56) in the control group (*t* = 7.17, *P* < 0.001), which showed statistical significance.

It was observed through comparison that the glycohemoglobin at 8 months of pregnancy, at 9 months of pregnancy, during labor, and 1 month after delivery was found to be remarkably lower in the experimental group than that in the control group (*P* < 0.05) (Figure 3).

### 3.5. Complications

**Figure 4:**
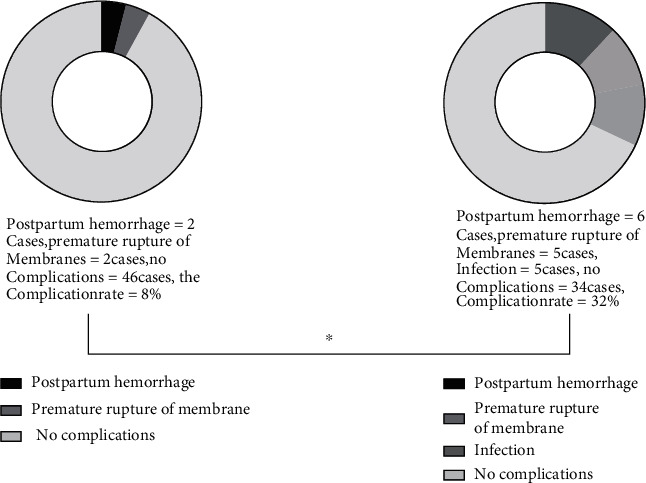
Comparison of complications between the two groups. (a) Displays the complications in the experimental group, including 2 cases of premature rupture of membranes and 2 cases of postpartum hemorrhage, with a complication rate of 8%. (b) Displays the complications in the control group, including 5 cases of premature rupture of membranes, 6 cases of postpartum hemorrhage, and 5 cases of infection, with a complication rate of 32%. ∗ indicates the comparison of the complication rate between the two groups (*X*^2^ = 9.00, *P* = 0.003), which was statistically significant.

It was noted by the comparison that the experimental group showed a lower incidence of complications in comparison with the control group (*P* < 0.05) (Figure 4).

### 3.6. CD3^+^, CD4^+^, and CD8^+^ Levels in Neonates

The immune function was judged by the determination of the CD3^+^, CD4^+^, and CD8^+^ levels in patients. It was found that the levels of CD3^+^, CD4^+^, and CD8^+^ were higher than in the experimental group as compared to the control group (*P* < 0.05) (Table 3).

## 4. Discussion

Gestational diabetes, one of the diseases specific to gestation that greatly threaten pregnant women and their fetuses, emerges due to hormonal disorders in pregnant women. Even the accumulation of sugar in vivo results in significantly high blood sugar or urine sugar [10–13]. For diabetes during pregnancy, given the tremendous effects of medication on the fetus, the control of glucose levels is mainly achieved through consistent and appropriate exercise and dietary control. Individualized nutritional intervention is utilized to reduce the risk arising from GDM by acting as a regulator of the nutritional input of patients to stabilize plasma sugar levels [14–17]. It was demonstrated in clinical trials that individualized nutritional intervention produced a promising effect on controlling and stabilizing blood sugar levels, indicating the great potential of individualized nutritional intervention in stabilizing plasma glucose in GDM patients [17, 18]. To conduct an intensive study on the effect of individualized nutritional intervention on gestational diabetic patients, patients with gestational diabetes were identified as research subjects in this study, and regular intervention and individualized nutritional intervention were performed, respectively, to analyze the nutritional status, labor outcomes, neonatal outcomes, complications, and neonatal immune function of patients.

The results of this study showed that the experimental group outperformed the control group with regard to natural labor rate, number of healthy newborns, maternal and neonatal MNA nutrition scores, and CD3^+^, CD4^+^, and CD8^+^ levels in newborns (*P* < 0.05). The main risks of gestational diabetes to the mother and fetus include hypertensive disorders in pregnancy, infection, excessive amniotic fluid, premature rupture of membranes, postpartum hemorrhage, fetal macrosomia, abnormal fetal development, preterm delivery, stillbirth, and neonatal hypoglycemia. The results of this study revealed that individualized intervention could reduce the incidence of macrosomia; therefore, individualized health education, psychological care, implementation of diet control, exercise therapy, and enhanced perinatal monitoring for pregnant women with gestational diabetes can effectively reduce obstetric complications and ameliorate maternal and infant outcomes. Moreover, the individualized nutritional intervention increased the natural labor rate. Cesarean section is a mode of delivery that has a great impact on the parturient and fetus. As it has a high mortality rate and may affect the reproductive potential of the mother, cesarean deliveries should be reduced to the greatest extent. Furthermore, natural labor such as normal childbirth is a crucial means of ensuring maternal and fetal health. Therefore, individualized nutritional intervention improves the rate of spontaneous labor onset and safeguards the health of the newborn and the mother. CD3^+^, CD4^+^, and CD8^+^ are indicators of immune function of the body. The levels of CD3+, CD4+, and CD8+ are correspondingly reduced in the presence of a weakened immune system and suppressed cellular immunity, exposing patients to a high risk of infectious diseases such as pathogenic infections [18, 19]. Herein, the immune function of newborns using individualized nutritional intervention was better than those receiving the conventional intervention. Currently, dietary control, exercise therapy, and insulin therapy are considered the three main methods for the treatment of gestational diabetes, of which dietary intervention is the most important, effective, and preferred method. Research has shown that the blood glucose levels of most patients with gestational diabetes are well managed through dietary control and all achieve favorable pregnancy outcomes [19]. The ideal standard of dietary control during pregnancy is to meet the nutritional needs of the gravidas and the fetus, to maintain stable weight gain, and to control blood glucose within normal limits without starvation ketosis. Intervention is primarily based on dietary guidance and reasonable exercise. With respect to their plasma glucose in the fasting state, 2 h after eating, and before bedtime; glycohemoglobin at 8 months of pregnancy, at 9 months of pregnancy, during labor, and 1 month after delivery; and their complications, they were obviously lower in the experimental group in relation to the control group (*P* < 0.05). Plasma glucose status 2 hours after meals and at bedtime is as significant as fasting glucose levels. Blood sugar at 2 h after meals may serve as an indicator of the presence of scientific diary structure in patients, whereas blood sugar before bedtime indicates the body's capability to bring down blood sugar levels. Individualized nutritional intervention was confirmed in this study to show a great significance to stabilize blood glucose, lower glycated hemoglobin levels, and decrease the incidence of complications in patients. Glycated hemoglobin was used to detect the stability of blood sugar in the past three months. If within the normal range, it indicates slight fluctuations in blood glucose in the short term and relatively stable blood glucose changes, which facilitates the stabilization of the function of the patient's organs to assist in lowering blood glucose. The research by Chen [20] suggested that early individualized nutritional intervention in GDM patients effectively lowers plasma glucose levels, indirectly improves gestational outcomes, and reduces the incidence of complications and adverse neonatal events, with significant prognostic value, which was consistent with those of the present study and fully demonstrated the scientific reliability of the results of this study. The limitations of this study are that the study was retrospective and biased, and future prospective randomized studies will be conducted, and interventions will be evaluated and given to pregnant women at different gestational stages.

To sum up, individualized nutritional intervention produces several effects in GDM patients such as increased rate of spontaneous delivery onset, enhanced immune function in their newborns, stable plasma glucose, and reduced complications. It possesses higher practicability in these patients, which merits clinical application.

## Figures and Tables

**Table 1:** Statistics of general data ($\bar{x}\pm s$).

Group	Experimental group	Control group	*P*
Age (Y)	27.68 ± 4.33	28.21 ± 4.29	0.54
Height (cm)	163.39 ± 8.87	162.88 ± 8.24	0.77
Prepregnancy weight	56.14 ± 3.31	55.98 ± 3.25	0.86
Body mass index (kg/m^2^)	25.59 ± 1.36	26.21 ± 1.96	0.94
Weight (kg)	68.62 ± 4.39	69.05 ± 4.55	0.63
Gestational age (W)	30.67 ± 2.30	30.41 ± 2.42	0.58
Fasting blood glucose	8.36 ± 2.11	8.40 ± 2.02	0.63
Hypertension (case)	10	8	0.60
Hyperlipidemia (case)	5	6	0.75
Smoking history (Y)	1.96 ± 0.33	2.00 ± 0.36	0.56
Alcohol history (Y)	4.22 ± 1.05	4.30 ± 1.02	0.70
First-born (case)	33	30	0.53
Non-first-born (case)	17	20	

**Table 2:** Comparison of neonatal outcomes between the two groups.

Group	Health	Congenital heart disease	Neonatal asphyxia	Fetal macrosomia
Experimental group	49	0	1	1
Control group	37	5	8	8
*X* ^2^	11.96	5.26	8.00	6.45
*P*	0.001	0.02	0.005	0.001

Health is considered if the newborn cries a few times after being born and then begin to breathe with the lungs at 40-50 times per minute for the first two weeks; the pulse of the newborn ranges from 120 to 140 beats per minute; the weight of the newborn ranges from 3000 to 4000 grams.

**Table 3:** Comparison of immune function between the two groups ($\bar{x}\pm s$, *μ*L^−1^).

Group	CD3^+^	CD4^+^	CD8^+^
Experimental group	904.49 ± 112.58	489.52 ± 88.07	480.17 ± 86.55
Control group	765.21 ± 101.39	361.25 ± 74.01	343.26 ± 73.91
*t*	6.50	7.88	8.51
*P*	<0.001	<0.001	<0.001

## Data Availability

No data were used to support this study.
